# Effect of internal jugular vein catheterization on intracranial pressure and postoperative cognitive function in patients undergoing robot-assisted laparoscopic surgery

**DOI:** 10.3389/fmed.2023.1199931

**Published:** 2023-05-04

**Authors:** Bin Yang, Min Li, Jingqiu Liang, Xixi Tang, Qi Chen

**Affiliations:** ^1^School of Clinical Medicine, Fujian Medical University, Fuzhou, Fujian, China; ^2^Department of Anesthesiology, The First Affiliated Hospital of Xiamen University, Xiamen, Fujian, China; ^3^Department of Anesthesiology, Chongqing University Cancer Hospital, Chongqing, China; ^4^Chongqing Cancer Multi-omics Big Data Application Engineering Research Center, Chongqing University Cancer Hospital, Chongqing, China

**Keywords:** internal jugular vein, optic nerve sheath diameter, postoperative delirium, robot-assisted laparoscopy, catheterization

## Abstract

**Background:**

We aimed to evaluate the effects of internal jugular vein (IJV) catheterization on intracranial pressure (ICP) and postoperative delirium (POD) during robot-assisted laparoscopic surgery by measuring the optic nerve sheath diameter (ONSD).

**Methods:**

Data from a prospective single-center cohort study, conducted from October 2021 to February 2022, were used. Forty out of 80 patients scheduled for laparoscopic radical hysterectomy or prostatectomy were assigned to the group receiving IJV catheterization (Group I), and the other 40 only received peripheral venous cannulation (Group C) according to clinical need of patients. Ultrasonography of ONSDs, the proportion of regurgitation time in a cardiac cycle, and hemodynamic parameters were measured at four time points: immediately after induction of anesthesia in the supine position (T0), 30 min (T1), 60 min (T2) after orienting to the Trendelenburg position, and before returning to the supine position at the end of surgery (T3). Time to eye opening and emergence stay, POD, and QoR-15 were compared.

**Results:**

The ONSDs increase gradually as the surgery progressed. Group I showed a higher value of ONSD at T1 (4.72 ± 0.29 mm vs. 4.5 ± 0.33 mm, *p* = 0.0057) and T3 (5.65 ± 0.33 mm vs. 5.26 ± 0.31 mm, *p* < 0.0001). The proportions of the regurgitation time of IJVV were greater in Group I than those in Group C at T1 (14.95, 8.5%–18.9% vs. 9.6%, 0%–17.2%, *p* < 0.0001) and T3 (14.3, 10.6%–18.5% vs. 10.4%, 0%–16.5%, *p* = 0.0003). Group I had a delayed time to eye opening (10.7 ± 1.72 min vs. 13.3 ± 2.35 min, *p* < 0.0001) and emergence stay (32.2 ± 5.62 min vs. 39.9 ± 6.7 min, *p* < 0.0001). There were no significant differences in POD and QoR-15 between the two groups on day three.

**Conclusion:**

IJV cannulation may not be the preferred approach in robot-assisted laparoscopic surgery as it was risk factor for IJVV regurgitation, ICP elevation, emergence delayed.

## 1. Introduction

Robot-assisted laparoscopic approach for major surgery offers several advantages including minimally surgical trauma, less blood loss, and faster recovery (1–3). However, the Trendelenburg position and CO_2_ pneumoperitoneum required for such surgery are highly associated with significant changes in cerebral hemodynamic physiology and intracranial pressure (ICP) (4–6). Due to its superficial location and relative security, the internal jugular vein (IJV) is usually the first choice for central venous puncture in these robot-assisted surgeries. However, catheterization of the IJV may impair the IJV valve (IJVV), which can cause IJVV incompetence (IJVVI) (7) and persistent valvular regurgitation and aggravate intracranial hypertension. Sustained elevation of ICP may cause postoperative delirium (POD) (8). Optic nerve sheath diameter (ONSD), as assessed by ultrasound, has shown sufficient correlation with real-time evaluation of ICP (9–11). Unfortunately, the effect of IJV catheterization on ICP and POD during robot-assisted laparoscopy has not been extensively investigated.

We hypothesize that IJV catheterization may aggravate ICP, manifested as an increase in ONSD, during robot-assisted laparoscopy, and this may be associated with the quality of emergence from anesthesia and incidence of POD.

## 2. Methods

This study was approved by the Institutional Review Board at Chongqing University Cancer Hospital (approval no. CZLS2021041-A) and registered prior to patient enrollment at clinicaltrials.gov (ChiCTR2100050863). After obtaining written informed consent, we enrolled patients scheduled for radical prostatectomy or hysterectomy via the da VinciTM robot system (Intuitive Surgical, Inc., Sunnyvale, CA, USA) between December October 2021 and February 2022. The exclusion criteria were as follows: (1) history of neurological disorder; (2) ocular trauma or orbital oedema; (3) cerebrovascular disease; (4) younger than 30 or older than 75; or (5) Delirium Screening scale (Nu—DESC) (12) ≥ 1 before the operation.

### 2.1. Interventions

Depending upon the requirement for postoperative IV nutrition, 40 patients underwent IJV catheterization (Group I) while the other 40 received peripheral venous cannulation (Group C). In Group I, The internal jugular vein catheter was inserted at 10–12 cm. After the catheterization operation, it was found through ultrasound observation that all the catheters crossed the internal jugular vein valve, except two patients with internal jugular vein valve absence.

Electrocardiography (ECG), peripheral arterial oxygen saturation (SpO2), and invasive arterial blood pressure of each patient were monitored. For the induction of general anesthesia, 0.4 ug/kg sufentanil, 2 mg/kg propofol, and 0.1 mg/kg vecuronium were used. The general anesthesia was maintained with a 1–1.5 minimum alveolar anesthetic concentration (MAC) sevoflurane and a target infusion concentration of 2–4 ng/ml remifentanil. Sufentanil and vecuronium were administered intermittently when required and BIS value maintained between 45–55. Respiratory management was performed with the help of pressure-controlled mechanical ventilation to maintain the airway pressure at 25 cmH_2_O in each patient. The respiratory rate was adjusted to maintain an end-expiratory carbon dioxide partial pressure (PETCO_2_) of 40–45 mmHg throughout the surgery.

### 2.2. Ocular sonography

When the patients were placed in a supine position after the administration of general anesthesia and at the other time points specified, a thick layer of gel was meticulously applied to the closed upper eyelid. The optimal image of the retrobulbar echogenic fat tissue and the vertical hypoechoic band was obtained by adjusting the angle of the linear probe without any excessive pressure (Mintray super 9 ultrasound, 7.5 MHz, depth of field: 4 cm). In addition, the ONSD was recorded as up to 3 mm behind the optic disc by focusing on the retrobulbar area. Measurements were taken in the transverse direction in both eyes, and the mean values of both measurements at each time point were used in the analysis.

### 2.3. IJVV regurgitation

The ultrasound scan was carried out in the B-mode with the short-axis view. A linear probe (Mintray super 7 ultrasound, 7.5 MHz, depth of field: 5 cm) was placed on the neck of each patient to visualize the IJV and carotid artery clearly, in a transverse position. The longitudinal axis view of the IJV was obtained by rotating the probe to 90 °. Intraluminal, curvilinear, mobile, and echoic structures were interpreted as a valve leaflet signal. The valve was usually located in the “J1” at segment of the IJV.

The Doppler flow velocity waves were created by freezing the images and its screenshot was obtained at the end of the expiratory phase. A line highlighting the mean regurgitant blood velocity can automatically overlap the Doppler blood velocity wave. Then, the proportion of regurgitation time in an average of three cardiac cycles was calculated.

### 2.4. Data collection and outcomes

Ultrasonographic measurement of ONSD and the proportion of regurgitation time of the right IJVV were primary outcomes which were conducted by an experienced anesthesiologist at the following distinct time points: immediately after induction of anesthesia in the supine position (T0), 30 min after achieving the Trendelenburg position (T1), 60 min after achieving the Trendelenburg position (T2), and before returning to the supine position at the end of surgery (T3).

The quality of emergence from general anesthesia was recorded, including time to eye opening and emergence stay, that is, the time from the end of surgery to eye opening or returning to the ward. Delirium was evaluated by a team of trained nurses using Nu—DESC twice a day until 3 days after the surgery. The quality of postoperative recovery was evaluated by the Quality of Recovery-15 (QoR-15) questionnaire on postoperative day 3. The above postoperative related indicators are the secondary outcomes of this study.

### 2.5. Statistical analysis

In the pre-test results, the mean ± standard deviation ONSD was 5.2 ± 0.4 mm at the end of surgery before returning to the supine position during robot-assisted laparoscopic surgery.

Assuming that the ONSD with IJV catheterization was increased by 5% as compared with the ONSD without IJV catheterization, the mean difference value of the ONSD between the two groups was 0.26 mm. Using a power of 90% and a significance level of *p* < 0.05, the sample size was calculated to be at least 35 subjects for each group. Considering a dropout rate of 10%, a total of 80 patients were enrolled in the study.

All continuous variables were either expressed as the mean ± standard deviation or as a number (%) if the standard of normally distributed data was met and medians and interquartile range (total range) if not. Comparisons between the groups of age, weight, height, ASA grade and QoR-15 scores, were performed using Student’s t-test if the standard of normally distributed data was met, and the Mann–Whitney test if not. A two-way repeated-measures analysis of variance with the Bonferroni post-test was used to compare the ONSD. Fisher exact test was used for incidence of POD and proportion of regurgitation time. *p*-values <0.05 were considered significant. SPSS version 18.0 (SPSS Inc., Chicago, IL, USA) was used for statistical analyses and depository. Figures were drawn using GraphPad Prism 8.0 (GraphPad Software, La Jolla, California, USA).

## 3. Results

**Figure 1:**
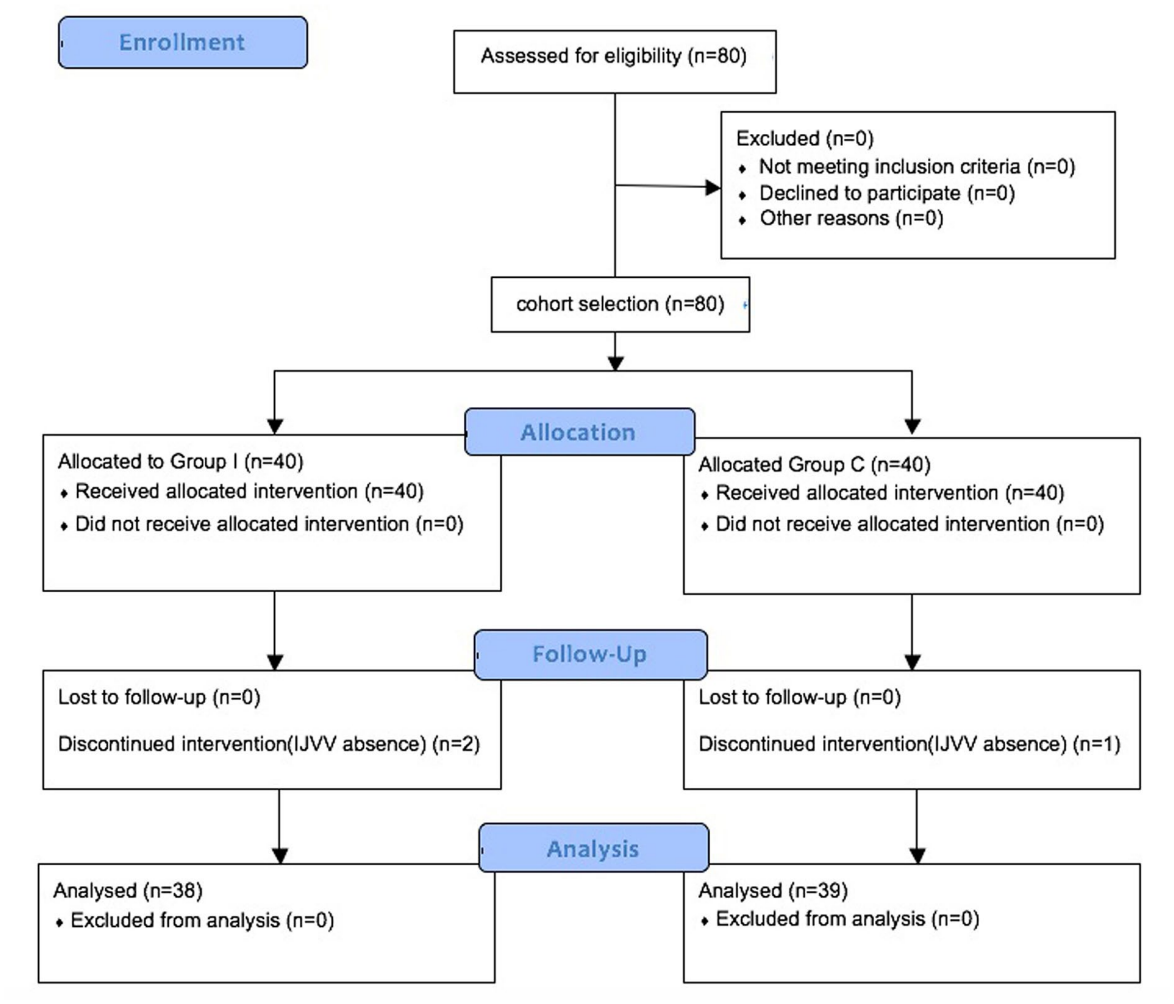
CONSORT flow diagrams showing the number of patients at each phase of the study.

**Figure 2:**
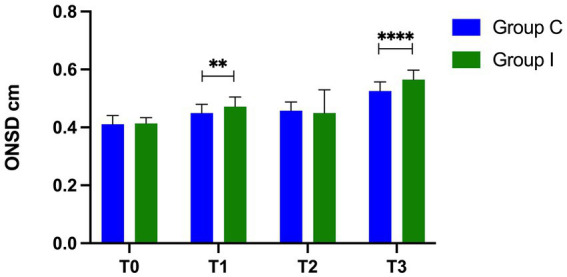
ONSD measurements at different time point. **means *p* <0.01, ****means *p* <0.0001, there is statistical significance between two groups.

**Figure 3:**
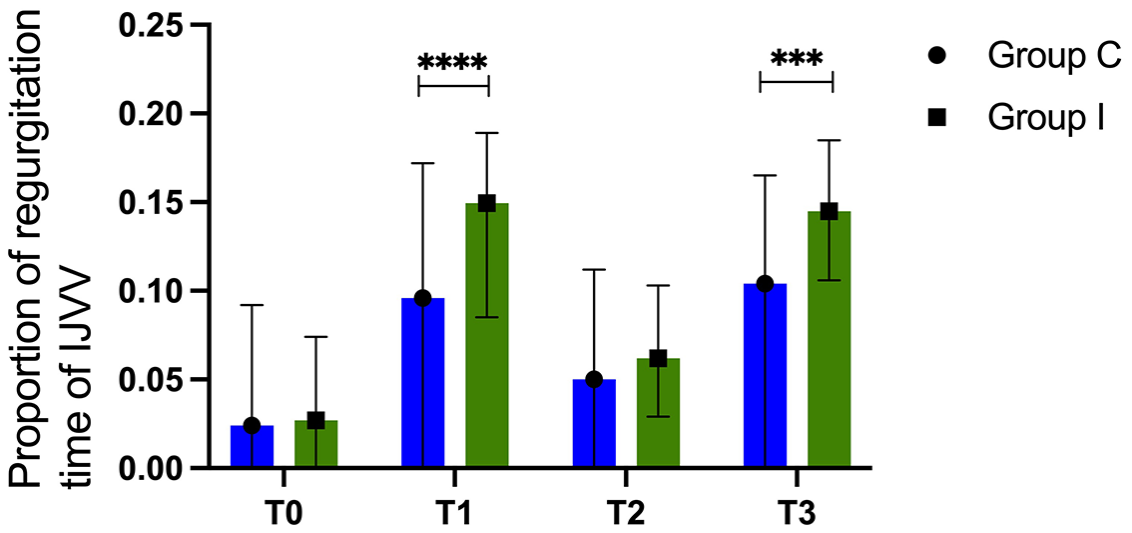
Proportion of regurgitation different time points. ***means *p* <0.001, ****means *p* <0.0001, there is statistical significance between two groups.

A total of 80 patients were screened for eligibility. After excluding three patients for the absence of IJVV detection during ultrasonography, 77 patients completed the study (Figure 1). There were no significant differences in demographic data (Table 1). Intraoperative variables of vital sigh and cardiopulmonary parameters were comparable (Table 2). The value of ONSD in T0 is comparable between Group I and Group C, and there is a gradual increase in the value of ONSD from T1 to T3 in both groups. Furthermore, the increase in Group I was more striking than that in Group C at T1 (4.72 ± 0.29 mm vs. 4.5 ± 0.33 mm, *p* = 0.0057) and T3 (5.65 ± 0.33 mm vs. 5.26 ± 0.31 mm, *p* < 0.0001; Figure 2). Compared with Group C, Group I had higher percentages of the proportion of regurgitation time through IJVV at T1 (14.95, 8.5%–18.9% vs. 9.6%, 0%–17.2%, *p* < 0.0001) and T3 (14.3, 10.6%–18.5% vs. 10.4%, 0%–16.5%, *p* = 0.0003)(Figure 3). Group I had a longer time to eye opening (10.7 ± 1.72 min vs. 13.3 ± 2.35 min, *p* < 0.0001) and emergence stay (32.2 ± 5.62 min vs. 39.9 ± 6.7 min, *p* < 0.0001). There was no significant difference in POD (0% vs. 2.6%, *p* = 0.493) and QoR-15 between groups (124.4 ± 9.3 vs. 122.9 ± 8.8, *p* = 0.464; Table 3).

**Table 1:** Characteristic of included patients.

Variables	Group I (*n* = 38)	Group C (*n* = 39)	*p* value
Age, y	55.9 ± 12.5	53.0 ± 11.5	0.297
Height, cm	161.1 ± 6.74	160.2 ± 6.1	0.569
Weight, kg	58.7 ± 7.74	57.6 ± 8.1	0.523
Gender (male/female)	8/30	6/33	0.119
Education, y	11.5 (9–12)	12 (9–15)	0.512
ASA physical status (I/II/III)	5/25/8	7/22/10	0.088
Surgery time, min	148 (120–163)	140 (122–158)	0.285
Hypertension	13	12	0.656
Diabetes mellitus	6	8	0.322
Thyroid disease	5	4	0.736

**Table 2:** Intraoperative variables.

	Group I (*n* = 38)	Group C (*n* = 39)	*p* value
MBP (mmHg)			
T0	77.6 ± 8.5	76.4 ± 8.8	0.452
T1	72.5 ± 8.1	72.2 ± 8.0	0.664
T2	73.6 ± 8.2	72.8 ± 7.8	0.304
T3	70.5 ± 7.7	71.1 ± 7.1	0.683
HR (beats/min)			
T0	84.5 ± 10.5	82.8 ± 10.2	0.223
T1	68.6 ± 7.9	70.3 ± 8.2	0.509
T2	67.4 ± 7.6	68.3 ± 7.9	0.687
T3	73.6 ± 8.6	74.7 ± 8.9	0.476
ETCO2 (mmHg)			
T0	34.6 ± 3.6	34.1 ± 3.5	0.464
T1	36.5 ± 4.0	35.7 ± 3.7	0.276
T2	38.6 ± 4.1	38.0 ± 3.9	0.397
T3	43.9 ± 5.2	43.5 ± 4.9	0.156
SV (mL)			
T0	46.2 ± 7.3	46.7 ± 7.8	0.638
T1	56.6 ± 9.0	55.4 ± 8.3	0.211
T2	55.3 ± 9.2	55.9 ± 8.8	0.302
T3	54.5 ± 8.0	54.1 ± 7.8	0.633
SVR (BSA)			
T0	1,205.5 (303)	1,163 (287)	0.330
T1	1,512.5 (398)	1,489 (366)	0.669
T2	1,455.5(328)	1,412 (311)	0.890
T3	1,402.5 (321)	1,356 (302)	0.621

**Table 3:** Perioperative index.

Variables	Group I (*n* = 38)	Group C (*n* = 39)	*p*
Time to eye open (min)	13.3 ± 2.36	10.7 ± 1.72*	<0.0001
Time for emergence stay (min)	39.9 ± 6.71	32.2 ± 5.69*	<0.0001
QoR-15	122.9 ± 8.6	124.4 ± 9.3	0.464
Incidence of POD	1 (2.6%)	0 (0%)	0.493

*p < 0.05 shows statistical significance between the two groups.

## 4. Discussion

The present study showed there is a gradual increase in ONSD and the proportion of regurgitation time of IJVV with surgical duration in robot-assisted laparoscopic surgery. The patients who received IJV cannulation had higher ICP at T3 which may be the reason for delayed postoperative emergence. However, there were no differences in POD and QoR-15 between the groups on day 3.

The IJVV, as the only valve between the thoracic and intracranial cavity, could prevent retrograde venous blood flow back to the brain (13, 14). Transient physiological regurgitation could be detected in normal competent IJVV (15). Pneumoperitoneum and the Trendelenburg position increased intrathoracic pressure and IJV catheterization could impair the function of IJV valves, thus aggravating the regurgitation (7, 16). Consistent with this thought, the proportion of regurgitation time of IJVV was increased at T1 and T3 in Group I. The neurological deficits associated with IJVV incompetence are thought to be caused by cerebral hypoperfusion resulting from increased ICP by brain venous congestion. Cerebrovascular autoregulation plays a significant role in maintaining the stability of blood flow and cerebral perfusion pressure (17, 18), but if it is disturbed, arterial hypertension may induce cerebral hyperemia and brain edema, while low arterial blood pressure can lead to cerebral ischemia.

Several previous studies have demonstrated excellent specificity and sensitivity between the ONSD and ICP, so intraoperative ICP changes can be evaluated by monitoring the ONSD (19, 20). The cut-off value of the ONSD corresponding to intracranial hypertension (ICP > 20 mmHg) in previous studies was 5 mm (21, 22). Our research showed that regardless of whether or not the IJV catheterization was performed, the ONSD reached more than 5 mm before returning to the supine position at the end of surgery. Furthermore, the values of the ONSD at T3 were significantly greater in patients with IJV catheterization, which means that both timing and catheterization both important factors.

Significant differences were found in the time to eye opening and emergence stay between patients with or without IJV catheterization. This may suggest the increase in ONSD and the proportion of regurgitation time of IJVV can potentially predict delayed emergence, excluding the effects of anesthetic drug residues and neuromuscular blockade. Although the conclusive definition of POD is not clear, it frequently occurred around 7 days in the early postoperative period, especially in the elderly with a 15% to 25% incidence after major surgery (23). In the present study, there were no differences in Nu—DESC and QoR-15 between the two groups, which means POD and the quality of postoperative recovery were comparable and indicates the adverse effects of IJV catheterization persist only for a short period. Fortunately, transient cerebral hypoperfusion is usually benign, but because it can cause cognitive dysfunction over prolonged period, it remains a potential risk.

Therefore, during robot-assisted laparoscopic surgery, we can distinguish those who may experience more severe ICP elevation with IJV catheterization. Moreover, anesthesiologists could choose the subclavian vein or the femoral vein as alternatives to the internal jugular vein if necessary.

There are some limitations in the study. First, POD was collected up to 3 days after surgery, so we may not detect the association between IJV catheterization and long-term neurological complications after surgery. It is important to note that POD can be newly developed 3 months after surgery, especially in the elderly (24). Second, only female patients were selected due to the inclusion of surgical indications and there is a lack of research data on male patients.

In conclusion, IJV cannulation may not be the preferred approach in robot-assisted laparoscopic surgery as it was a risk factor for IJVV regurgitation, ICP elevation, delayed emergence.

## Data availability statement

The original contributions presented in the study are included in the article/supplementary material, further inquiries can be directed to the corresponding author.

## Ethics statement

The studies involving human participants were reviewed and approved by Institutional Review Board at Chongqing University Cancer Hospital. The patients/participants provided their written informed consent to participate in this study.

## Author contributions

BY contributed to the study design, data analysis, and manuscript writing. ML contributed to patient recruitment and ultrasound procedures. JL contributed to ultrasound procedures and patient recruitment. XT contributed to statistical analysis and data collection. QC is the guarantor of the integrity of the entire study and manuscript review. All authors contributed to the article and approved the submitted version.

## Funding

This work was supported by Chongqing medical scientific research project (Joint project of Chongqing Health Commission and Science and Technology Bureau 2023MSXM125), Natural Science Foundation of Fujian Province (2022 J011369), Xiamen Medical and Health Guidance Project (3502Z20224ZD1023), Clinical project of fujian university of traditional Chinese Medicine (XB2021087 to Bin Yang), and Fujian Research and Training Grants for Young and Middle-aged Leaders in Healthcare.

## Conflict of interest

The authors declare that the research was conducted in the absence of any commercial or financial relationships that could be construed as a potential conflict of interest.

## Publisher’s note

All claims expressed in this article are solely those of the authors and do not necessarily represent those of their affiliated organizations, or those of the publisher, the editors and the reviewers. Any product that may be evaluated in this article, or claim that may be made by its manufacturer, is not guaranteed or endorsed by the publisher.
